# Use of Diode Laser in Hysteroscopy for the Management of Intrauterine Pathology: A Systematic Review

**DOI:** 10.3390/diagnostics14030327

**Published:** 2024-02-02

**Authors:** Andrea Etrusco, Giovanni Buzzaccarini, Antonio Simone Laganà, Vito Chiantera, Salvatore Giovanni Vitale, Stefano Angioni, Maurizio Nicola D’Alterio, Luigi Nappi, Felice Sorrentino, Amerigo Vitagliano, Tommaso Difonzo, Gaetano Riemma, Liliana Mereu, Alessandro Favilli, Panagiotis Peitsidis, Antonio D’Amato

**Affiliations:** 1 Unit of Obstetrics and Gynecology, “Paolo Giaccone” Hospital, 90127 Palermo, Italy; etruscoandrea@gmail.com; 2 Department of Health Promotion, Mother and Child Care, Internal Medicine and Medical Specialties (PROMISE), University of Palermo, 90127 Palermo, Italy; vito.chiantera@unipa.it; 3 Department of Obstetrics and Gynecology, IRCCS San Raffaele Scientific Institute, Vita-Salute San Raffaele University, 20132 Milan, Italy; giovanni.buzzaccarini@gmail.com; 4 Unit of Gynecologic Oncology, National Cancer Institute IRCCS Fondazione “G. Pascale”, 80131 Naples, Italy; 5 Division of Gynecology and Obstetrics, Department of Surgical Sciences, University of Cagliari, 09124 Cagliari, Italy; salvatoreg.vitale@unica.it (S.G.V.); sangioni@yahoo.it (S.A.); mauridalte84@gmail.com (M.N.D.); 6 Department of Medical and Surgical Sciences, Institute of Obstetrics and Gynecology, University of Foggia, 71121 Foggia, Italy; luigi.nappi@unifg.it (L.N.); felice.sorrentino.1983@gmail.com (F.S.); 7 Department of Interdisciplinary Medicine (DIM), Unit of Obstetrics and Gynecology, University of Bari “Aldo Moro”, Policlinico of Bari, Piazza Giulio Cesare 11, 70124 Bari, Italy; amerigo.vitagliano@gmail.com (A.V.); difonzo.tommaso.md@gmail.com (T.D.); antoniodamato19@libero.it (A.D.); 8 Department of Woman, Child and General and Specialized Surgery, University of Campania “Luigi Vanvitelli”, 80138 Naples, Italy; gaetano.riemma7@gmail.com; 9 Unit of Obstetrics and Gynecology, Department of General Surgery and Medical-Surgical Specialism, University of Catania, P.O. “G. Rodolico”, Via Santa Sofia, 78, 95123 Catania, Italy; liliana.mereu@unict.it; 10 Section of Obstetrics and Gynecology, Department of Medicine and Surgery, University of Perugia, 06135 Perugia, Italy; alessandro.favilli@unipg.it; 11 Department of Obstetrics and Gynecology, Helena Venizelou Hospital, 11521 Athens, Greece; peitsidiobgyn@gmail.com

**Keywords:** diode laser, hysteroscopy, hysteroscopic surgery, endometrial polyps, fibroids, septate uterus

## Abstract

**Background:** Hysteroscopy currently represents the gold standard for the diagnosis and treatment of intrauterine pathologies. Recent technological progress has enabled the integration of diagnostic and operative time, leading to the “see and treat” approach. Diode laser technology is emerging as one of the most innovative and intriguing techniques in this context. **Methods:** A comprehensive search of the literature was carried out on the main databases. Only original studies reporting the treatment of intrauterine pathologies using diode laser were deemed eligible for inclusion in this systematic review (PROSPERO ID: CRD42023485452). **Results:** Eight studies were included in the qualitative analysis for a total of 474 patients undergoing laser hysteroscopic surgery. Eighty-three patients had female genital tract abnormalities, 63 had submucosal leiomyomas, 327 had endometrial polyps, and one patient had a scar pregnancy. Except for leiomyomas, whose technique already included two surgical times at the beginning, only seven patients required a second surgical step. Cumulative rates of intraoperative and postoperative complications of 2.7% and 0.6%, respectively, were reported. **Conclusions:** Diode laser through “see and treat” hysteroscopy appears to be a safe and effective method. However, additional studies with larger sample sizes and improved designs are needed to consolidate the evidence currently available in the literature.

## 1. Introduction

### 1.1. Background

Lasers represent an alternative energy source to electrosurgery that is gaining interest in gynecologic surgery [1,2,3,4]. Several types of lasers have been used in the gynecologic field: the Nd-Yag laser, Argon laser, CO_2_ laser, and the newer diode laser [5,6]. For a laser to be suitable for endoscopic use, it must possess four characteristics: clean-cutting ability, good hemostatic effect, superficial tissue penetration, and release through optical fibers. However, some of the currently available lasers have some shortcomings: the CO_2_ laser cannot be delivered with fibers but with articulated mirror arms, and the Nd-YAG laser, due to its low wavelength absorbing water, has a higher risk of deep tissue penetration than other lasers.

Diode is an electronic laser consisting of two very dim semiconductor materials. A microprocessor regulates the flow of electric current through the diode to generate the laser beam. Once generated, the beam is transmitted through an optical system to an optical fiber, which acts as a carrier for the light carried to the point of operation. The wavelengths produced can range from 980 to 1470 nm. Due to these wavelengths, the diode laser achieves high simultaneous absorption by hemoglobin and water, providing hemostatic properties and thus offering high ablation and vaporization capabilities [7,8]. The diode laser results in significantly greater hemostasis than does the less-modern CO_2_ laser, and its thermal penetration is lower than that of the Nd-YAG laser, enabling the implementation of a precise and safe procedure [9,10,11,12]. Its use can be extended to laparoscopic or hysteroscopic gynecologic surgery.

Historically, hysteroscopy was conceived as a diagnostic procedure designed to reach directly into the uterine cavity and visualize its contents. Years after its introduction, it is now considered the gold standard for the diagnosis and treatment of intrauterine pathologies, such as endometrial polyps, submucosal myomas, uterine abnormalities, or postsurgical outcomes such as intrauterine synechiae or isthmoceles [13,14,15,16,17,18].

The development of hysteroscopes with features increasingly tailored to the characteristics of the cervical canal has not only facilitated the transition of procedures from the operating room to the outpatient clinic but also helped reduce patient discomfort and made this technique increasingly appreciated and used worldwide [19,20]. This shift has also made it possible to combine diagnostic and operative time, giving rise to the modern “see and treat” approach, which has tangible benefits in terms of reducing the number of procedures performed and improving overall patient satisfaction [21,22,23,24].

Currently, the availability of instruments for treating intrauterine conditions, both in inpatient and outpatient settings, is more than extensive, synthesizing the latest electronic technologies to the ability to miniaturize them to make them suitable for this type of endoscopic surgery [25,26]. Laser technology was adapted and integrated into hysteroscopy by experimenting with different types of lasers, such as the Nd-Yag laser [27,28,29,30], the potassium titanyl phosphate (KTP) laser [31], or the argon laser [32]. Recently, the use of diode lasers in hysteroscopy has increased in various operative settings [33]. 

### 1.2. Objectives

The aim of this systematic review was to evaluate the use of diode laser for “see-and-treat” hysteroscopy in the management of intrauterine pathology.

## 2. Methods

### 2.1. Eligibility Criteria

Only original studies (retrospective or prospective) reporting the treatment of uterine and endometrial pathologies using diode laser were deemed eligible for inclusion in this systematic review. We included both studies with patients desiring offspring and studies with menopausal patients. Due to the lack of qualified reviews related to the main topic of our paper, case reports or case series with fewer than 10 patients were also considered suitable for inclusion in the qualitative analysis to fill this gap.

Studies describing only the technology used without reporting outcomes by pathology and studies describing only the procedure technique (described step-by-step procedure) were excluded.

Likewise, studies on other laser types or non-English language studies were not considered eligible for inclusion in this systematic review.

### 2.2. Information Sources

This systematic review (PROSPERO ID: CRD42023485452) was carried out according to the Preferred Reporting Items for Systematic Reviews and Meta-Analyses (PRISMA) guidelines [34] and validated by the Enhancing the Quality and Transparency of Health Research (EQUATOR) network and the Cochrane Handbook for Systematic Reviews [35].

MEDLINE, EMBASE, Global Health, The Cochrane Library (Cochrane Database of Systematic Reviews, Cochrane Central Register of Controlled Trials, Cochrane Methodology Register), Health Technology Assessment Database, Web of Science and Research Register (ClinicalTrial.gov) were searched for studies describing surgical procedures for uterine and endometrial pathologies using diode lasers.

### 2.3. Search Strategy

The following medical subject heading (MeSH) and key search terms were used for each database: “Hysteroscopy” (MeSH Unique ID: D015907), “Hysteroscopic surgery” (MeSH Unique ID: D015907), “Diode laser” AND “Leiomyoma” (MeSH Unique ID: D007889), “Uterine Anomalies” (MeSH Unique ID: C562565), “Endometrial polyps” (MeSH Unique ID: D011127), “Uterine Synechiae” (MeSH Unique ID: D006175), and “Isthmocele”. We selected papers written in English from the inception of each database until 30 November 2023.

### 2.4. Study Selection

Titles and/or abstracts of studies retrieved using the search strategy were screened independently by two review authors (A.E. and A.S.L.) to identify studies that met the inclusion criteria. The full texts of these potentially eligible articles were retrieved and independently assessed for eligibility by two other review team members (A.D. and G.B.). A manual search of the references of the included studies was also conducted to prevent the omission of pertinent research. Any disagreement between the reviewers over the eligibility of the articles was resolved through discussion with a third (external) collaborator. All authors approved the final selection.

### 2.5. Data Extraction

Two authors (A.E. and A.D.) independently extracted data from articles about study features, characteristics of the included populations, surgical procedures, complications and results/outcomes using a pre-piloted standard form to ensure consistency. One author (A.S.L.) reviewed the entire data extraction process.

### 2.6. Assessment of Risk of Bias

Two reviewers (A.E. and A.D.) independently assessed the risk of bias in the studies included in this systematic review using a modified version of the Newcastle-Ottawa Scale (NOS) [36]. The quality of the studies was evaluated in the following five different domains: “study design and sample representativeness”, “sampling technique”, “description of the hysteroscopic technique”, “quality of the population description”, and “incomplete outcome data” (Table S1). Any disagreements between the reviewers were resolved by a third reviewer (G.B.).

### 2.7. Outcome Measures and Data Synthesis

The primary objective of this study was to evaluate the efficacy, safety, and feasibility of diode laser in the treatment of intrauterine pathology, as described below.

- “Efficacy”: efficacy was measured by the success rate of the procedures, as determined by the absence of residual lesions at the end of the procedure and/or at the follow-up visit.
- “Feasibility”: feasibility was assessed as the rate of procedures completed in a single surgical step, without interruptions due to surgical problems or patient complaints.
- “Safety”: safety was determined by the rate of intraoperative and postoperative complications.

Quantitative analysis was not possible due to data heterogeneity (including different settings and surgical procedures). We provided a descriptive synthesis of the results in separate sections based on the type of pathology that was hysteroscopically removed or corrected: polyps, leiomyomas, female genital tract anomalies, and cesarean scar pregnancy.

The body of evidence on the usefulness of diode lasers for each pathology was assessed by two authors (A.E., A.S.L.) using the Oxford Centre for Evidence-Based Medicine 2011 Levels of Evidence (OCEBM) [37].

## 3. Results

### 3.1. Study Selection

**Figure 1:**
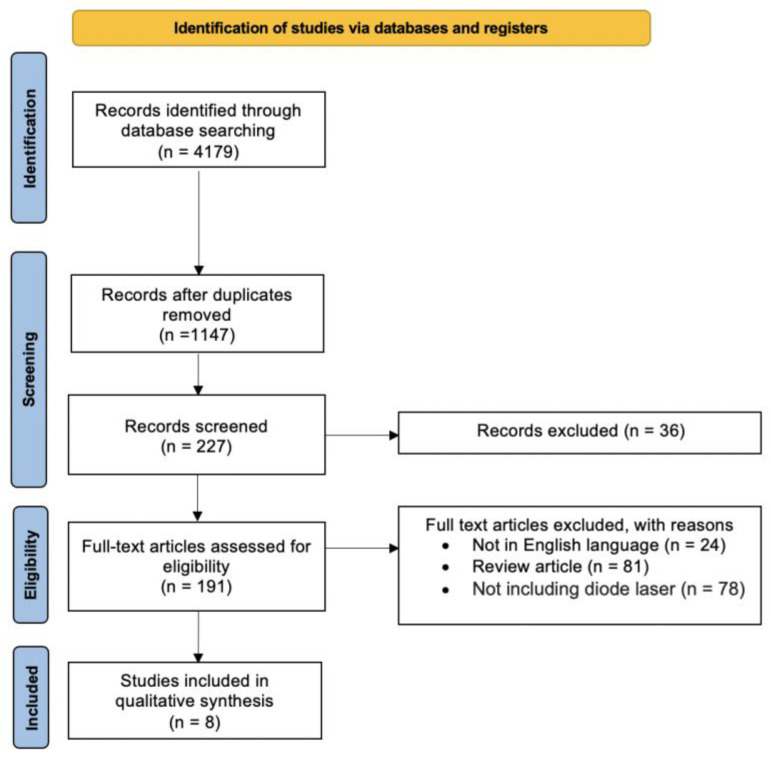
PRISMA flow diagram of the review.

The study selection process is displayed in Figure 1. After the evaluation of the full texts, a total of eight papers [9,10,11,38,39,40,41,42] that met the abovementioned inclusion criteria were included in the present systematic review.

### 3.2. Study Characteristics

The main characteristics of the included studies are summarized in Table 1. Only one retrospective study included prospective follow-up [11]. The other seven studies were prospective and included one randomized controlled trial (RCT) [9], three pilot studies [38,39,40], one multicenter prospective cohort study [10], one prospective cohort study [42] and one case report [41]. Of these, four studies were from Italy [39,40,41,42], three from Spain [9,10,38] and one from Israel [11].

### 3.3. Risk of Bias of Included Studies

Among the eight studies included, seven had a low risk of bias in three or more domains [9,10,11,38,39,40,42], and only one was judged to have a high risk of bias [41]. A detailed description of the risk of bias in each domain among the studies is reported in Table S2.

### 3.4. Synthesis of the Results

Among the included studies, three evaluated the use of diode laser for female genital tract anomalies [10,11,39], two for the treatment of uterine leiomyomas [38,42], two for endometrial polyps [9,40], and one for cesarean scar pregnancy [41]. As previously mentioned, we discussed the results separately based on the type of uterine pathology treated in the various included studies.

#### 3.4.1. Female Genital Tract Anomalies

Congenital malformations of the female genital tract deviate from normal anatomy as a result of altered embryological development of Müllerian ducts [43]. The type and degree of anatomical distortion are associated with health and reproductive problems and can cause repeated miscarriages and infertility [44]. For these reasons, they are a common indication for hysteroscopy [45].

Three studies evaluated the correction of female genital tract anomalies using diode laser during hysteroscopic metroplasty [10,11,39] and were included in the present analysis. In two of those studies, the main objective was to assess the effectiveness and feasibility of hysteroscopic metroplasty with diode laser for the septate uterus [10,39], while in the other study, the use of laser technology was employed for the correction of a dysmorphic uterus [10].

In chronological order, the first publication was a pilot study by Nappi et al. [39]. Eighteen patients with V-b class septate uteri according to the American Society of Reproductive Medicine (ASRM) guidelines [46] or with Class U2a septate uteri according to the European Society of Human Reproduction and Embryology (ESHRE)–European Society for Gynecological Endoscopy (ESGE) classification [47] were included. Of these patients, 11 suffered from recurrent pregnancy loss (RPL), and seven from primary infertility. All procedures were conducted by two operators using a 5 mm Bettocchi hysteroscope with a vaginoscopic approach in an outpatient setting. A polyfiber was inserted through the 5 Fr working channel of the hysteroscope and further connected to a 980 nm wavelength laser device, set to 20 W of power in continuous mode. Intraoperative pain was assessed using a visual analog scale (VAS) ranging from 0 to 10. The operation time was 13.16 ± 1.33 min, and the incidence of intraoperative pain was 3.05 ± 0.72. All procedures were performed successfully, and no intraoperative or postoperative complications occurred. Follow-up hysteroscopy was carried out for all patients 2 months post hysteroscopic metroplasty, and no intrauterine adhesions or recurrence of the septum was diagnosed. All patients underwent postsurgical follow-up for 6–30 months, and reproductive outcomes were evaluated. The clinical pregnancy rate was 63.6% (7/11) in the RPL group and 71.4% (5/7) in the infertility group. One patient in the infertility group experienced a spontaneous abortion (14.3%), and eventually, six live births occurred.

Similar results, although with a greater need for a second surgery (*n* = 0 vs. *n* = 7), were obtained by Esteban Manchado et al. [10].

Finally, a retrospective cohort study with prospective follow-up [11] was included in the present systematic review. Twenty-five patients with dysmorphic uteri underwent hysteroscopic metroplasty via a vaginoscopic approach under general anesthesia. The mean age was 35.4 ± 5.4 years. All of them had a diagnosis of infertility, RPL, or recurrent implantation failure (RIF). Following three-dimensional (3D) ultrasound (US), 15 patients were diagnosed with a T-shaped uterus (the U1a class according to the ESHRE-ESGE classification) [47], and 10 patients were diagnosed with a Y-shaped uterus, which is a subtype of T-shaped uterus according to several authors [48]. All procedures were performed by the same surgeon with a 5 mm Bettocchi hysteroscope. A conical 1000-micron probe was introduced in the 5 Fr operative channel and connected to a 1470 nm wavelength diode laser device set to 15 W of power for the procedures. The mean duration of the procedure was 25 ± 7 min. No intraoperative or postoperative complications occurred. Among the 25 treated patients, 15 subsequently underwent in vitro fertilization (IVF) treatment. Nine clinical pregnancies (60%) and two miscarriages (13.3%) occurred; among the nine clinical pregnancies, the authors reported seven with a live birth or an ongoing pregnancy. Additional data regarding the characteristics of the included studies are listed in Table 2.

**Quality of evidence:** The evidence regarding the safety, effectiveness, and reliability of diode laser for the correction of female genital tract anomalies was classified as level 3.

#### 3.4.2. Uterine Leiomyomas

Uterine leiomyomas are benign monoclonal smooth muscle cell tumors of the myometrium [49] and represent the most common pathology of the female genital tract [50]. Although most myomas are asymptomatic, some, depending on their location, size, and number, can be responsible for pelvic pain, abnormal uterine bleeding, and states of subfertility and infertility [51,52]. Two studies examined the application of diode laser for treating uterine leiomyomas [38,42].

The first, in chronological order, was a pilot study by Haimovic et al. [38], which sought to evaluate the viability of a novel two-step approach for office hysteroscopic resection of submucous myomas. Forty-three women diagnosed with a single, symptomatic G1 or G2 myoma according to the 2005 ESGE classification [53] and a size of less than 4 cm were consecutively enrolled. All patients were of reproductive age. Prior to surgery, all patients underwent a transvaginal ultrasound examination to assess the location, size, and type of submucous myoma. All of the procedures were performed by the same operator with a 4 mm Bettocchi hysteroscope. Overall, the technique involved a modified variant of the OPPIuM approach described by Bettocchi’s group [54], with substantial modifications, such as the use of diode laser instead of conventional bipolar energy, and the performance of both surgical steps in an outpatient setting with the patient awake and without the use of anesthesia. Full enucleation was successfully accomplished in 80.9% (17/21) of the patients with G1 myomas and 77.3% (17/22) of those with G2 myomas (*p* = 1.000). In total, 34 patients successfully underwent the two-step myomectomy procedure, accounting for 79.1% of the total cohort. The median (interquartile range, IQR) duration of the initial step was 16 min, while the second step required a median time of 24 min. The mean VAS scores for the first and second office hysteroscopic steps were 2.49 ± 0.83 and 3.07 ± 1.01, respectively. Patients, on average, expressed a satisfaction score of 3.51 ± 1.06, with 75% reporting a satisfaction level equal to or greater than 3. Patients were not followed up for evaluation of reproductive outcomes.

The second study was a prospective cohort study [42] involving 20 patients diagnosed with a single, symptomatic myoma of less than 7 cm in size according to the 0–2 grade of the FIGO PALM-COEIN classification [55] who were undergoing hysteroscopic laser ablation (HLA) of the tumor. All of the patients were fertile, had a desire for offspring, and suffered from heavy menstrual bleeding. Seventy percent of patients were diagnosed with FIGO G1 myoma, whereas the remaining 30% were diagnosed with fibroids classified as G2. HLA was performed with a 3.8 mm Bettocchi hysteroscope via vaginoscopy in an outpatient setting without the use of anesthesia. A 1470 nm diode laser device set to 15 W of energy was connected to a probe, which was introduced into the operative channel of the hysteroscope. After confirming the correct position of the tip of the probe at the center of the fibroid, continuous circular energy was applied, and the fibroid edges were coagulated. The procedure was concluded when the surgeon observed sufficient coagulation of the fibroid. The mean operation duration was 7.0 ± 2.1 min. The mean intraoperative pain duration following VAS assessment in all patients was 2.9 ± 2.0. Eighty-eight percent of patients (18/20) showed marked improvement in menstrual bleeding at 2 months post procedure (*p* = 0.001). All of the patients were monitored by 3D-US 2 months after the procedure, and the mean reduction in fibroid volume 2 months post procedure was 36%. Reproductive outcomes after surgery were not subsequently assessed. Table 3 provides supplementary information on the included research studies.

**Quality of evidence:** The evidence regarding the safety, effectiveness, and reliability of diode laser for the treatment of leiomyomas was classified as level 3.

#### 3.4.3. Endometrial Polyps

Endometrial polyps are localized tumors of the endometrial mucosa [56]. As a common cause of abnormal uterine bleeding [55] and even infertility [57,58], they represent a common indication for hysteroscopy [1]. Two studies evaluated the feasibility of diode laser energy for the hysteroscopic removal of endometrial polyps [9,40].

Among these, the first trial was an RCT by Lara-Domínguez [9], in which 102 patients with suspected US endometrial polyps were randomized to undergo polypectomy either with a bipolar electrode (Versapoint) or with diode laser. In the Versapoint group, hysteroscopic polypectomy was carried out with a 5 mm Bettocchi hysteroscope via a vaginoscopic approach without anesthesia. A bipolar electrode was inserted through the 5 Fr operative channel of the instrument. On the other hand, in the diode laser group, the procedure was performed through a 6 mm hysteroscope with a 7 Fr operative channel, through which a polyfiber connected to a 980 nm laser device was inserted. Intraoperative VAS scores were assessed for all patients in both groups. Complete successful transection of the polyp was achieved in 96.1% of the Versapoint group and in 92.0% of the diode laser polypectomy group. The mean time required for polyp resection using the diode laser was significantly shorter than that required for polyp resection using the Versapoint technique (245.96 ± 181.9 s vs. 329.56 ± 245.0 s, respectively; *p* = 0.01). Furthermore, no differences were detected in intraoperative pain levels (VAS score 4.4 ± 2.9 vs. 4.4 ± 2.9, *p* = 0.91) assessed using the VAS or an ordinary pain level scale ranging from 0 to 10. For all patients, a follow-up hysteroscopy was scheduled 3 months after the first hysteroscopy. Eleven patients were lost to follow-up for various reasons. During the second hysteroscopic examination, recurrence of the polyp at the same site occurred in 15 patients (32.6%) in the Versapoint group. In contrast, in the diode laser group, polyp recurrence was noted in only one patient (2.2%) (*p* = 0.001). Before the second hysteroscopy, patients were asked to complete questionnaires regarding their degree of satisfaction with the procedure, the impact of the procedure on their quality of life, and whether they would recommend the procedure. The rates of “very satisfied” and “highly recommendable” patients were significantly greater in the diode laser group than in the Versapoint group.

The second trial was a pilot study [40] that included 300 women with a suspected US diagnosis of endometrial polyps. A total of 225 patients who eventually met the inclusion criteria underwent hysteroscopic polypectomy with a 4 mm Bettocchi hysteroscope. Two distinct fiber types were employed: a 1000 μm Bare Fiber with a Ball Tip and a 715 μm Bare Fiber with a Conical Tip, connected to a dual-wavelength laser system to generate a 980 + 1470 nm laser through diode semiconductor. Following the “see and treat” approach, 97.3% of the patients underwent successful laser polypectomy. The VAS score and procedural duration were positively correlated with polyp size, with higher VAS scores and longer procedural times associated with larger polyps. Additional details about the included studies are presented in Table 4.

**Quality of evidence:** We found adequate quality evidence (level 2) supporting the effectiveness, feasibility, and safety of using diode lasers for outpatient endometrial polypectomy.

#### 3.4.4. Cesarean Scar Pregnancy

Cesarean scar pregnancy (CSP) represents one of the rarest forms of intrauterine pregnancy [59,60] and occurs when the implant is located in the scar of a previous cesarean section. Only one article described the use of diode laser for the treatment of CSP.

Sorrentino et al. [41] reported the treatment of CSP with a combined two-step radiological and endoscopic gynecological approach. A 40-year-old woman with one prior vaginal delivery and two previous cesarean sections presenting with 5.5-week amenorrhea was admitted to the Department of Obstetrics and Gynecology exhibiting an abrupt onset of scant vaginal bleeding accompanied by mild lower abdominal cramping. A transvaginal US scan revealed a CSP. The first step of the treatment involved uterine artery embolization by cannulation of the right femoral artery and subsequent injection of a gelatin hemostatic sponge in both uterine arteries. The day after the radiological procedure, the patient underwent operative hysteroscopy via a 3.8 mm Bettocchi hysteroscope via vaginoscopy without anesthesia. A 980 nm and 1470 nm dual-wave laser device was connected to a conical angled fiber, through which the laser excision of the ectopic pregnancy was performed. The patient had a regular postoperative recovery and was discharged after 3 days. After 4 weeks of follow-up, the patient was in good condition and asymptomatic.

**Quality of evidence:** The evidence regarding the safety, effectiveness, and reliability of diode lasers for the treatment of CSP was classified as level 5.

## 4. Discussion

In 1981, Milton Goldrath introduced what can be considered the inaugural hysteroscopic endometrial ablation, utilizing an Nd:YAG laser [61]. Since this pioneering procedure, laser vaporization has gained global acceptance. Unfortunately, its integration into gynecological surgery has been hindered by prohibitive costs, limited availability, and challenging learning curves [5]. Despite these constraints, laser technology has evolved with noteworthy advantages [1,2,3,4].

Laser technology, where LASER denotes “Light Amplification by Stimulated Emission of Radiation”, operates by amplifying a specific wavelength of light, generating a photon beam. When the laser beam contacts organic tissue, it induces molecular vibrations, leading to the breakage of chemical bonds and the production of heat [5,8]. This enables precise cutting, controlled tissue vaporization, regulated penetration power, high hemostatic capability, and safety, all achieved without the need for anesthesia.

A crucial advantage of this technology is the absence of electrical interference. Surgeons have favored laser technology over bipolar energy due to the latter’s association with thermal damage, impacting both the sample for histopathological examination and the adjacent healthy tissue [62,63]. In recent years, various lasers, including argon, krypton, Nd:YAG, and diode lasers, have been successfully employed. However, the Nd:YAG laser stands out as the most widely applied in hysteroscopic procedures [64].

The evolution of hysteroscopy from its origins as a primary diagnostic tool to its status as the gold standard for both the diagnosis and treatment of intrauterine pathologies represents an exciting journey in gynecological practice [65]. The integration of hysteroscopes tailored to cervical canal shape and conformation has not only facilitated the shift from operating rooms to outpatient clinics, but also significantly enhanced patient comfort [66,67,68]. This evolution has given rise to the contemporary “see and treat” approach, in which diagnostic and operative procedures are effectively combined, leading to a reduction in the number of interventions and an enhancement in overall patient satisfaction [69]. The “see and treat” approach cannot be separated from adequate training in diagnostic hysteroscopy; diagnostic hysteroscopy must be critically imparted to future endoscopists so that they understand whether the pathology they face can be addressed on an outpatient basis or whether they should defer treatment to address it in an operative setting [70,71,72,73]. In this context, diode laser technology represents a powerful tool in the hands of an expert endoscopist for the treatment of a wide range of endocavitary diseases, as shown in previous studies. 

This new type of laser, with a power of 15 W and a wavelength of 1470 nm, works only in contact with a dispersion heat of 0.5–1 mm, with minimal damage to surrounding tissues [10,11] and, as shown by the present qualitative analysis, it has already been safely and successfully applied in the treatment of intrauterine pathologies.

Owing to its use in hysteroscopic metroplasty, laser technology has shown good safety and reliability, as well as promising results in terms of reproductive outcomes [10,11,39]. Future studies with a control group, preferably employing a randomized surgical approach between the two groups, are needed to assess the actual impact of diode lasers on infertility and reproductive outcomes in affected patients compared to conventional surgical techniques. Haimovic et al. [38] demonstrated the feasibility of using laser energy in combination with the two-step approach initially introduced by Bettocchi’s group [54] for treating FIGO G1-2 myomas. This highlights new possibilities for its prospective application in uterine myoma treatment. Future studies should evaluate whether the application of diode lasers can be extended to FIGO G3 myomas, as their reclassification to submucosal uterine fibroids [74] should also make hysteroscopic treatment the gold standard treatment [75,76,77,78]. Moreover, Vitale et al. [42] described a completely different approach in which diode laser was utilized for coagulation and thermoablation of the core of the fibroid, with favorable results. The implementation and increased diffusion of laser technology among surgeons could contribute to elevating the percentage of patients treated with fibroids through a “see and treat” approach in an outpatient setting. This approach has the potential to diminish waitlists and costs for hospitals while concurrently reducing patient discomfort and the need for anesthetic medications. From this perspective, the use of diode laser in hysteroscopy is among the most innovative minimally invasive techniques for treating uterine fibroids and is comparable to other methods, such as high-intensity focused ultrasound, microwaves, and radiofrequency ablation [79,80,81,82]. Nevertheless, this approach finds fertile ground like no other approach in the management of endometrial polyps. Lara- Domínguez et al. conducted a RCT [9] with rigorous methodology and post-treatment hysteroscopic follow-up. Their findings suggest that, despite similar intraoperative pain levels, diode lasers in hysteroscopic endometrial polypectomy may even outperform conventional bipolar energy in terms of the duration of the procedure, recurrence rate, and patient satisfaction. Furthermore, the technique finds extensive application in a see-and-treat context, as demonstrated in the series by Nappi et al. [40], where more than 200 patients were treated using the same approach, with excellent results in terms of surgical outcome and low VAS scores. Finally, although not supported by solid literature, another minimally invasive method that is increasingly moving hysteroscopic surgery from the operating room to the outpatient setting is the mini-resectoscope. The mini-resectoscope is a widely used instrument in the hysteroscopic treatment of intrauterine pathology, and its miniaturization would seem to bring with it all the advantages of the conventional resectoscope, together with a procedure that is quicker and well tolerated by the patient and thus conducted without the aid of anesthesia in most cases [83,84,85,86,87,88,89]. Given the increasing use of laser and mini-resectoscope technology in “see and treat” hysteroscopy, future studies should be conducted with the aim of verifying the superiority of one technology over the other in terms of efficacy and safety.

To the best of our knowledge, this is the first systematic review investigating the efficacy, safety, and feasibility of treating intrauterine pathology with the diode laser. 

Despite the use of the diode laser by experienced gynecologic endoscopists worldwide, the evidence gathered is limited, with only eight studies published to date. Although it was possible to highlight low the intra- and postoperative complication rates (2.7% and 0.6%), as well as low need for reintervention (1.7%, excluding patients with myoma, for whom two surgical steps were planned from the beginning), it was not possible to obtain sufficient information to assess reproductive outcomes for patients desiring offspring.

In addition, reports of “step-by-step procedure descriptions” according to the pathology treated are also not available in the literature. Although they do not contribute to the accumulation of evidence, they may be of great help to the reader from both scientific and clinical perspectives.

Finally, it is important to recognize that the level of evidence varied significantly according to the pathology treated, from 2 to 5.

## 5. Conclusions

Diode laser technology can be considered a safe and effective hysteroscopic treatment for intrauterine pathology through a “see and treat” outpatient approach, reducing waitlists and costs, enhancing patient comfort, and minimizing the need for anesthesia. In the field of minimally invasive techniques for the treatment of uterine diseases, diode lasers in hysteroscopy align with other innovative technologies and set the stage for possible future widespread use. Future studies should be conducted with larger sample sizes and improved designs to consolidate the evidence currently available in the literature.

## Figures and Tables

**Table 1:** Characteristics of the included studies.

Author	Year	Type	Main Outcome	Country	Patient (*n*)	Age (Mean)	Control Group	Participant Characteristics	Intervention	Registration
Haimovic et al. [38]	2013	Pilot	To evaluate the feasibility of a new two-step technique for office hysteroscopic resection of submucous myoma	Spain	43	36.7	none	Reproductive-age patients with symptomatic lesions diagnosed sonographically as single G1 or G2 myoma ≤ 4.0 cm	Two-step hysteroscopic procedure: preparation of partially intramural myomas by incision of the endometrial mucosa and pseudocapsule covering the myoma in the first step, and excision of the myoma by diode laser 4 weeks later.	None
Lara-Domínguez et al. [9]	2015	Randomized Controlled Trial	To compare the resection of endometrial polyps using Versapoint bipolar electrode versus diode laser	Spain	102	51.5	yes	Patients with endometrial polyps, single or multiple	Hysteroscopic diode laser polypectomy	Clinical Trial ID: NCT02126397
Nappi et al. [39]	2016	Pilot	To evaluate the feasibility and safety of office hysteroscopic metroplasty using a 980 nm diode laser	Italy	18	32.7	none	Patients with sonographically diagnosed endometrial polyps ≤ 2.5 cm	Hysteroscopic diode laser polypectomy	None
Nappi et al. [40]	2016	Pilot	To evaluate the feasibility and effectiveness of hysteroscopic endometrial polypectomy using a new dual wavelength laser system	Italy	300	54	none	Patients with V-b or Class U2a septate uterus, in according with ASRM guidelines and the ESHRE-ESGE classification	Hysteroscopic diode laser metroplasty after 14-day endometrial preparation with 5 mg per day of nomegestrol acetate	None
Esteban Manchado et al. [10]	2020	Multicenter Prospective Cohort Study	To investigate the effectiveness and safety of office hysteroscopic metroplasty by diode laser for the treatment of septate uteri	Spain	41	34.2	none	Women diagnosed with V-b or Class U2a septate uterus, in accordance with ASRM guidelines and the ESHRE-ESGE classification, and a history of primary infertility or recurrent miscarriage	Hysteroscopic diode laser metroplasty	None
Sorrentino et al. [41]	2021	Case Report	To report a case of cesarean scar pregnancy treated by combined uterine artery embolization and hysteroscopic laser surgery	Italy	1	40	none	40-year-old woman with cesarean scar pregnancy	Angiographic uterine artery embolization followed by hysteroscopic diode laser resection	None
Bilgory et al. [11]	2021	Retrospective Cohort Study	To study the efficacy and safety of diode laser hysteroscopic metroplasty for dysmorphic uterus and the impact on reproductive outcomes	Israel	25	35.4	none	Nulliparous woman with T- or Y-shape uterus and infertility	Hysteroscopic diode laser metroplasty	None
Vitale et al. [42]	2023	Prospective Cohort Study	To evaluate the feasibility and efficacy of in-office hysteroscopic ablation of submucous uterine fibroids using diode laser	Italy	20	39.1	none	Patients with at least one symptomatic, class 0–2 FIGO classification, uterine fibroids ≤ 7 cm in size.	Laser vaporization of the fibroid core	Clinical Trial ID: NCT05604001

ASRM: American Society for Reproductive Medicine; ESHRE: European Society of Human Reproduction and Embryology, ESGE: European Society for Gynecological Endoscopy; FIGO: International Federation of Gynecology and Obstetrics.

**Table 2:** Summary of studies evaluating the use of diode laser in hysteroscopic metroplasty for the correction of female genital tract anomalies.

	Nappi et al. [39]	Manchado et al. [10]	Bilgory et al. [11]
**Patients (*n*)**	18	40	25
Mean age (years)	32.66 ± 2.74	34.2 ± 5.278	35.4 ± 5.4
BMI (kg/m^2^)	21.58 ± 1.63	n.d.	25.4 ± 5.4
**Symptoms**			
Infertility (%)	38.9	37.5	n.d.
RPL (%)	61.1	62.5	n.d.
RIF (%)	n.d.	n.d.	n.d.
**Female genital tract anomaly**			
Type of uterine anomaly	Septate uterus	Septate uterus	Dysmorphic uterus (T-shape and Y-shape)
ASRM/ESHRE Class	Vb–U2a	Va–U2b	U1a
**Preoperative assessment**			
Preoperative 3D-US (%)	100	100	100
**Surgery**			
Mean operative time (min)	13.16 ± 1.33	- First step: 12.74 ± 3.552 - Second step: 9.85 ± 1.345	25 ± 7
Surgeon (*n*)	2	n.d.	1
Mean VAS	3.05 ± 0.72	2.225 ± 0.5768 (1 to 5)	n.d.
Intraoperative complications (*n*)	0	0	0
Postoperative complications (*n*)	0	1	0
Adhesions (*n*)	0	1	0
Need for a surgical second step (*n*)	0	7	0
**Follow-up**		1	0
Mean follow-up time (months)	6–30	24	11.5 ± 9.2
Postoperative follow-up hysteroscopy (%)	100	100	100
**Reproductive outcomes**			
Clinical pregnancy rate before surgery (%)	n.d.	n.d.	33.3
Clinical pregnancy rate after surgery (%)	- Primary infertility group: 71.4 - RPL group: 63.6	78.9	60
Miscarriage rate before surgery (%)	n.d.	n.d.	40
Miscarriage rate after surgery (%)	- Primary infertility group: 14.28 - RPL group: 0	20	13.3
Live birth rate before surgery (%)	n.d.	n.d.	0
Live birth rate after surgery (%)	- Primary infertility group: 42.9 - RPL group: 27.3	63.2	46.7

BMI: body mass index; RPL: recurrent pregnancy loss; RIF: recurrent implantation failure; ASRM: American Society for Reproductive Medicine; ESHRE: European Society of Human Reproduction and Embryology; 3D-US: 3D ultrasound; VAS: visual analog scale; n.d.: not declared.

**Table 3:** Summary of studies evaluating the use of diode laser for the treatment of uterine leiomyomas.

	Haimovic et al. [38]	Vitale et al. [42]
**Patients (*n*)**	43	20
Mean age (years)	36.7 ± 4.6	39.1 ± 4.7
BMI (kg/m^2^)	n.d.	21.4 ± 1.6
Fertile age	100	100
Mean parity	0.79 ± 0.94	n.d.
**Symptoms**		
Abnormal menstrual bleeding (%)	44.2	90
Pelvic pain (%)	11.6	60
Infertility (%)	44.2	n.d.
Increased urinary frequency (%)	n.d.	30
Bulking symptoms (%)	n.d.	70
**Preoperative assessment**		
Preoperative 3D-US (%)	100	100
**Characteristics of myomas**		
ESGE/FIGO class G1 (%)	48.8	70
ESGE/FIGO class G2 (%)	51.2	30
Mean size	21.7 ± 7.3	
**Localization of myomas**		
Anterior wall	46.5	n.d.
Posterior wall	30.2	n.d.
Fundus	16.3	n.d.
Lateral walls	7.0	n.d.
**Surgery**		
Technique used	Two-step hysteroscopic resection	Hysteroscopic laser ablation
Mean operative time (min)	- First step: 16 (IQR) - Second step: 24 (IQR)	n.d.
Surgeon (*n*)	1	1
Mean VAS	- First step: 2.49 ± 0.83 - Second step: 3.07 ± 1.01	2.9 ± 2.0
Intraoperative complications (*n*)	0	0
Postoperative complications (*n*)	0	0
Need for a surgical second step (*n*)	0	0
**Follow-up**		
Postoperative follow-up	n.d.	3D-US
Reproductive outcomes	n.d.	n.d.

BMI: body mass index; 3D-US: 3D ultrasound; ESGE: European Society for Gynecological Endoscopy; FIGO: International Federation of Gynecology and Obstetrics; VAS: visual analog scale; n.d: not declared.

**Table 4:** Summary of studies evaluating the use of diode laser for the treatment of endometrial polyps.

	Lara-Domínguez et al. [9]	Nappi et al. [40]
**Patients (*n*)**	102	225
Mean age (years)	- Diode laser group: 49.1 ± 10.3 - Versapoint group: 53.9 ± 10.2	54 ± 12.6
BMI (kg/m^2^)	- Diode laser group: 49.1 ± 10.3 - Versapoint group: 53.9 ± 10.2	26.55 ± 4.23
Fertile age (%)	41.2	38.7
Menopausal (%)	58.8	61.3
Mean parity	- Diode laser group: 2.1 ± 1.1 - Versapoint group: 2.0 ± 1.0	2.11 ± 1.71
**Symptoms**		
Asymptomatic (%)	- Diode laser group: 46 - Versapoint group: 44.2	n.d.
Hypermenorrhea (%)	- Diode laser group: 14.0 - Versapoint group: 7.7	n.d.
Metrorrhagia (%)	- Diode laser group: 40.0 - Versapoint group: 48.1	n.d.
**Preoperative assessment**		
Preoperative US (%)	100	100
**Characteristics of polyps**		
Mean size (mm)	21.7 ± 7.3	n.d.
Size 0–1 cm (*n*)	n.d.	94
Size 1–2.5 cm (*n*)	n.d.	131
**Localization of polyps (*n*)**		27
Anterior wall	n.d.	63
Posterior wall	n.d.	28
Fundus	n.d.	87
Lateral walls	n.d.	3
Isthmus	n.d.	17
Peri-ostial	n.d.	27
**Surgery**		
Mean operative time (min)	- Diode laser group: 245.96 ± 181.9 - Versapoint group: 329.56 ± 245.0	- Women in reproductive age, size of polyp 1–2.5 cm: 13 ± 0.90 - Menopausal women, size of polyp 0–1 cm: 9 ± 0.45
Surgeon (*n*)	2	n.d.
Mean VAS	- Diode laser group: 4.4 ± 2.9 - Versapoint group: 4.4 ± 2.9	Women in reproductive age: - 0–1 cm 1.773 ± 2.39 - 1–2.5 cm 2.054 ± 1.494 Menopausal women: - 0–1 cm 1.622 ± 1.803 - 1–2.5 cm 1.703 ± 2.271
Intraoperative complications (*n*)	3	6
Vagal syndrome/intolerance (*n*)	3	6
Incomplete resection of polyp	- Diode laser group: 4 - Versapoint group: 2	0
Postoperative complications (*n*)	1	0
Pelvic inflammatory disease (*n*)	1	0
**Follow-up**		
Postoperative follow-up (%)	Hysteroscopy: 89.2%	Ultrasound: 100%
Polyp relapse (%)	- Diode laser group: 2.2 - Versapoint group: 32.6	0
Very satisfied with the procedure (%)	- Diode laser group: 62.2 - Versapoint group: 39.1	n.d.
Highly recommendable procedure (%)	- Diode laser group: 71.1 - Versapoint group: 28.3	n.d.
**Reproductive outcomes**	n.d.	n.d.

BMI: body mass index; VAS: visual analog scale; n.d: not declared.

## Data Availability

Not applicable (no new data were generated during the development of this systematic review).

## Supplementary Materials

The following supporting information can be downloaded from https://www.mdpi.com/article/10.3390/diagnostics14030327/s1. Table S1: Modified Newcastle–Ottawa scale items; Table S2: Risk of bias assessment.
