# Supplementary material for: Use of Diode Laser in Hysteroscopy for the Management of Intrauterine Pathology: A Systematic Review
Source: Diagnostics (Basel). 2024 Feb 2;14(3):327. doi: 10.3390/diagnostics14030327 (PMC10855490; doi:10.3390/diagnostics14030327)
Supplement: Supplementary file 1 [file diagnostics-14-00327-s001.zip › Table S2.pdf]

Author	Study design and sample representativeness	Sampling technique	Description of the hysteroscopic technique	Quality of population description	Incomplete outcome data	Total score	Risk of bias
Lara-Dominguez et al. [9]	★	★	★	-	★	★★★★	Low
Esteban Manchado et al. [10]	-	-	★	★	★	★★★	Low
Bilgory et al. [11]	-	-	★	★	★	★★★	Low
Haimovic et al. [38]	-	★	★	★	★	★★★★	Low
Nappi et al. [39]	-	-	★	★	★	★★★	Low
Nappi et al. [40]	-	★	★	★	★	★★★★	Low
Sorrentino et al. [41]	-	-	★	-	★	★★	High
Vitale et al. [42]	-	-	★	★	★	★★★	Low

Table S2. Risk of bias assessment.
