# Supplementary material for: Use of Diode Laser in Hysteroscopy for the Management of Intrauterine Pathology: A Systematic Review
Source: Diagnostics (Basel). 2024 Feb 2;14(3):327. doi: 10.3390/diagnostics14030327 (PMC10855490; doi:10.3390/diagnostics14030327)
Supplement: Supplementary file 1 [file diagnostics-14-00327-s001.zip › Table S1.pdf]

**(1)**	**Study design and sample representativeness:**<br>1 point: Study design involved a control group, sample size was greater than or equal to 100 participants and exclusion rate was lower than 20%.<br>0 points: Uncontrolled study, sample size less than 100 participants or exclusion rate higher than 20%.
**(2)**	**Sampling technique:**<br>1 point: Patients recruited consecutively or randomly (randomization criteria clarified).<br>0 points: Potential convenience sampling or unspecified sampling technique.
**(3)**	**Description of the hysteroscopic technique:**<br>1 point: The authors provided a comprehensive description of the equipment, setting, and adopted technique.<br>0 points: The study did not report adequate information on the hysteroscopic technique.
**(4)**	**Quality of population description:**<br>1 point: The study reported a clear description of the population (e.g. age, BMI, duration of infertility, characteristics of the intrauterine lesion and surgical procedures, etc.) with proper measures of dispersion (e.g., mean, standard deviation).<br>0 points: The study did not report a clear description of the population, incompletely reported descriptive statistics, or did not report measures of dispersion.
**(5)**	**Incomplete outcome data:**<br>1 point: The study reported complete data about feasibility, efficacy and safety of the procedures and/or reproductive outcomes.<br>0 points: Selective data reporting cannot be excluded.

Table S1. Modified Newcastle-Ottawa scoring items.

The individual components listed above are summed to generate a total modified Newcastle-Ottawa risk of bias score for each study. Total scores range from 0 to 5.

For the total score grouping, studies were judged to be of low risk of bias (≥3 points) or high risk of bias (<3 points).
